# Application of Metabolomics in Childhood Leukemia Diagnostics

**DOI:** 10.1007/s00005-022-00665-6

**Published:** 2022-11-07

**Authors:** Agata Kozioł, Małgorzata Pupek

**Affiliations:** Department of Biochemistry and Immunochemistry, Medical University of Wroclaw, Wrocław, Poland

**Keywords:** Metabolomics, Cancer, Childhood leukemia, Nuclear magnetic resonance, Mass spectrometry, FT-IR

## Abstract

Metabolomics is a new field of science dealing with the study and analysis of metabolites formed in living cells. The biological fluids used in this test method are: blood, blood plasma, serum, cerebrospinal fluid, saliva and urine. The most popular methods of assessing the composition of metabolites include nuclear magnetic resonance spectroscopy and mass spectrometry (MS) in combination with gas chromatography–MS or liquid chromatography–MS. Metabolomics is used in many areas of medicine. The variability of biochemical processes in neoplastic cells in relation to healthy cells is the starting point for this type of research. The aim of the research currently being carried out is primarily to find biomarkers for quick diagnosis of the disease, assessment of its advancement and treatment effectiveness. The development of metabolomics may also contribute to the individualization of treatment of patients, adjusting drugs depending on the metabolic profile, and thus may improve the effectiveness of therapy, reduce side effects and help to improve the quality of life of patients. Here, we review the current and potential applications of metabolomics, focusing on its use as a biomarker method for childhood leukemia.

**Graphic abstract:**

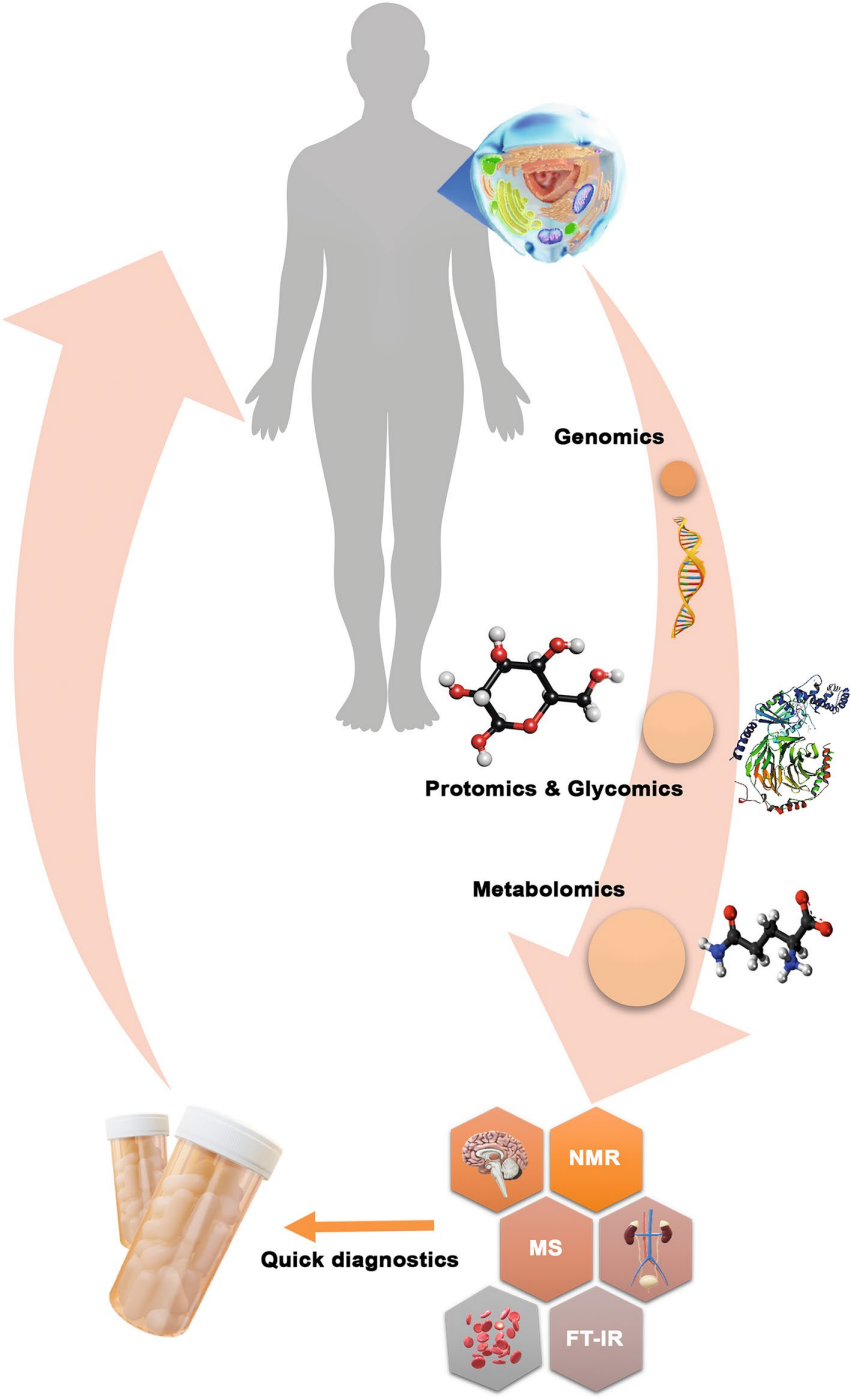

## Introduction

In the metabolism of living organisms, metabolites, i.e., low-molecular-weight organic compounds such as carbohydrates, organic acids, amino acids, nucleosides, nucleotides, etc., play a fundamental role. Together with macromolecules such as proteins and nucleic acids, metabolites play a variety of functions in the ongoing processes life (Brindle 2015).They can be of both endogenous and exogenous origin, e.g., drugs. It is assumed that endogenous chemical compounds released into body fluids are better determinants of the biological phenotype of an organism than gene expression (Dang et al. 2009). The metabolic profile takes into account not only the genetically determined phenotype, but also differences in the expression of phenotype traits conditioned by environmental factors such as diet, age, and physical fitness (Fiehn et al. 2000). This is a significant advantage of metabolomics over genomics, i.e., the analysis of genetic material. The study of the products of biological changes may lead to the broadening of knowledge about the processes taking place in the cell, and their quantification will enable the examination of the pathomechanisms of disease development and the search for potential early markers of disease (Clarke and Foster 2012; Dang et al. 2009).

**Fig. 1:**
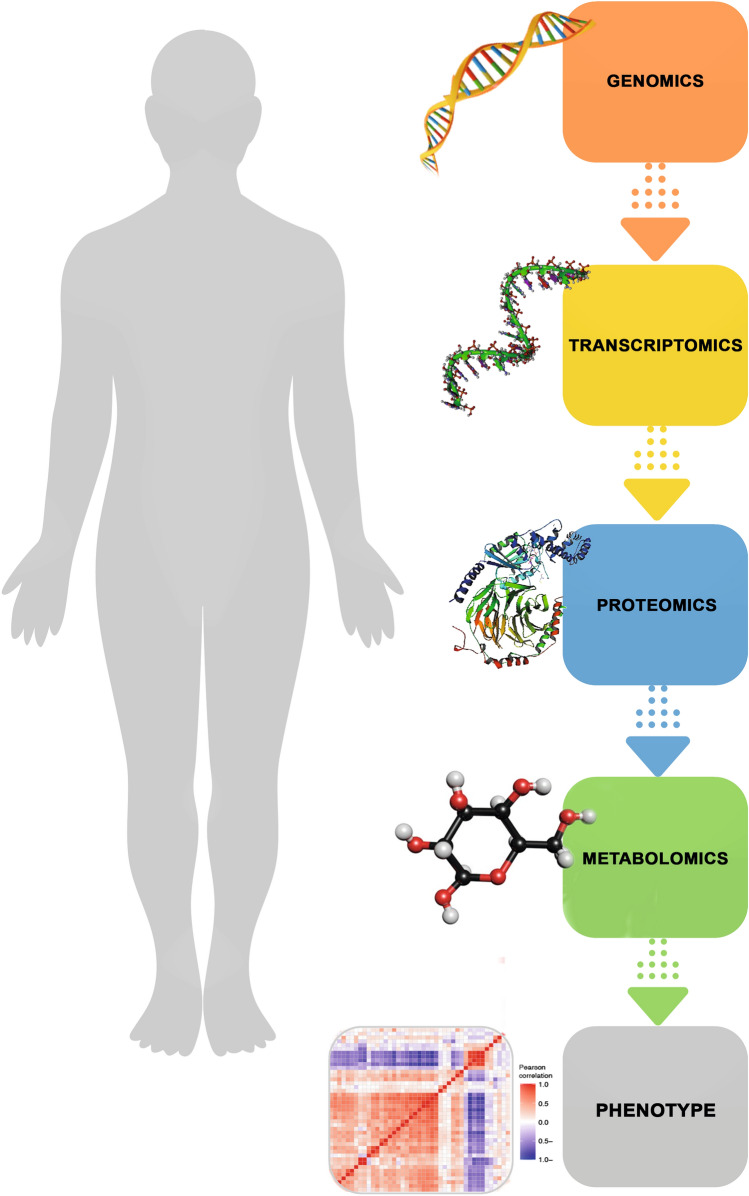
Overview of the central dogma, including fundamentals and design principles for multi-omics profiling in systems biology

The metabolites are most often extracted into aqueous or methanolic solutions. From these solutions, individual polar and lipophilic fractions are separated and analyzed. There is no single extraction method that is suitable for all types of metabolites. Conditions that are appropriate for one metabolite may degrade other types of metabolites or interfere with their analytical determination. For example, nucleotides (e.g., ATP) are extracted with very strong perchloric acid. As a strong oxidant, this acid causes the oxidation of other metabolites. Alkaloids are extracted in an alkaline medium which is destructive to aldehyde compounds. Thiol compounds, being very unstable, require the use of derivatizing reagents that transform them into stable derivatives, but at the same time prevent the analysis of other metabolites. Extraction methods and types must target a specific group of metabolites. So far, the metabolomics research has focused mainly on compounds of adequate stability that could be extracted together (carbohydrates, phosphate esters, amino acids, or organic acids). The most complete metabolomics analyses mentioned in the literature characterized up to 300 different compounds (Fig. 1). Naturally, many other relevant metabolites are ignored in this situation (Clarke and Foster 2012; Dang et al. 2009; Fiehn et al. 2000).

Contemporary research methods make it possible to use various biological materials for the analysis of the patient’s metabolome. The basic biological fluids used in metabolomics are: blood, blood plasma, serum, cerebrospinal fluid (CSF), saliva, and urine. Blood and urine have the greatest potential in detecting disease markers. Metabolomics, on the other hand, deals with the detection of low-molecular-weight substances that are present in relatively low concentrations and are not normally detected in basic blood tests. The metabolites in the blood are usually hydrophilic substances. Compared to other biological fluids, such as saliva or CSF, blood is characterized by a significantly greater number and variety of chemical compounds, including metabolites (Haug et al. 2013; Schraw et al. 2021). Thanks to the use of modern research methods, metabolites can be detected in the CSF in trace amounts. This makes it easier to find in the sample specific substances that may indicate pathological changes in the body. The research to date has been limited mainly to the determination of single, previously identified compounds or groups of metabolites. Progress in bioanalytical science made it possible to use advanced technologies, such as nuclear magnetic resonance (NMR) or mass spectrometry (MS), and this in turn forced the development of appropriate mathematical tools for processing the huge amounts of data obtained as a result of research. Thus, although studies of cellular metabolism or quantification of metabolic processes are not new in science, it was not until the end of the twentieth century that it became possible to perform a comprehensive analysis of virtually all metabolites found in cell fluids (Brindle 2015; Clarke and Foster 2012; van der Greef et al. 2004).

In this review, we will present metabolomics methodology and discuss how it is being applied in the field of oncology with particular attention to its application as a biomarker in childhood leukemia diagnosis, assessing treatment effects.

## Techniques for Metabolite Analysis

In metabolomics studies, due to the need to simultaneously determine a large number of metabolites, the currently most frequently used analytical technique is MS (Table 1). At the same time, separation techniques preceding MS are used to separate metabolites of the same molecular weight. The use of an extremely versatile detection method, such as MS, enables the simultaneous analysis of compounds on the basis of their interactions with the separation system as well as on the basis of the molecular weights of the analytes. Gas chromatography (GC) coupled to MS (GC–MS) requires sample derivatization (chemical modification) to increase the stability and volatility of the metabolites (Moldoveanu and David 2015). Subsequently, the chromatographically separated fractions of the derivatized sample are introduced into a mass spectrometer, where they are ionized and determined in high resolution on the basis of their mass-to-charge ratio. Using the GC–MS technique, Fiehn et al. (2000) were able to determine 326 different metabolites in Echinacea plant extracts. Only half of the marked metabolites could be identified. Identification of the remaining ones would theoretically be possible after using MS/MS or NMR techniques. The use of two-dimensional (2D) gas chromatography (GC/GC) in conjunction with MS is helpful for the separation of analytes originally unresolved in GC–MS (Table 2).

**Table 1:** Selected metabolites of melanoma, along with the corresponding detected parent ions, adducts and fragments of MS / MS, and theoretical and experimental m / z

Name	Adducts	Theoretical m/z	Experimental m/z	ESI
8-Hydroxy-deoxyguanosine or Guanosine	M-H	282.084	282.0839	(–)
Malic acid	M-H	133.014	133.0146	(–)
7-Hydroxy-6-methyl-8-ribityl lumazine	M + K-2H	365.051	365.0495	(–)
β-Citryl-l-glutamic acid	M-H	320.062	320.0621	(–)
S-pyruvyl glutathione	M + FA-H	422.088	422.0868	(–)
Oxidized glutathione	M-H	611.145	611.1432	(–)
3-Phenylserine	M + H	182.0812	182.0812	( +)
Methionine	M + H	150.0582	150.0582	( +)
Phenylalanine	M + H	166.0862	166.0862	( +)
Niacinamide	M + H	123.055	123.0550	( +)

*m/z*—mass-to-charge ratio, *ESI*—electrospray ionization

**Table 2:** Selected marker metabolites found in GC / MS chromatograms

Metabolite	*R*_*t*_ (min)	Chemical class	Metabolite identification using standard compound
Lactate	9.85	Organic acid	Standard
Phosphate	14.65	Inorganic acid	Standard
*L*-Glycine	15.40	Amino acid	Standard
*L*-Proline	22.37	Amino acid	Standard
*L*-Phenylalanine	23.06	Amino acid	Standard
*D*-Galactose	31.00, 32.38	Monosaccharide	Standard
*D*-Glucose	32.16, 32.41, 34.00	Monosaccharide	Standard
Arachidonic acid	41.71	Fatty acid	Standard
Cholesterol	53.70	Steroid	Standard

Two-dimensional GC/GC combines chromatographic columns of different polarity and analyzes at different temperatures so as to diversify the retention of previously unresolved metabolites as much as possible (Zhang et al. 2014).

Another technique for the initial separation of sample components is high-performance liquid chromatography coupled with MS (LC–MS). Unlike GC–MS, LC–MS samples generally do not derivate prior to analysis. The separation of the metabolites into the individual fractions takes place on a chromatographic column. The LC–MS technique is more commonly used than GC–MS, because it is more suitable for unstable, difficult to derivatize or non-volatile compounds (Deglon et al. 2012; Haug et al. 2013).

**Fig. 2:**
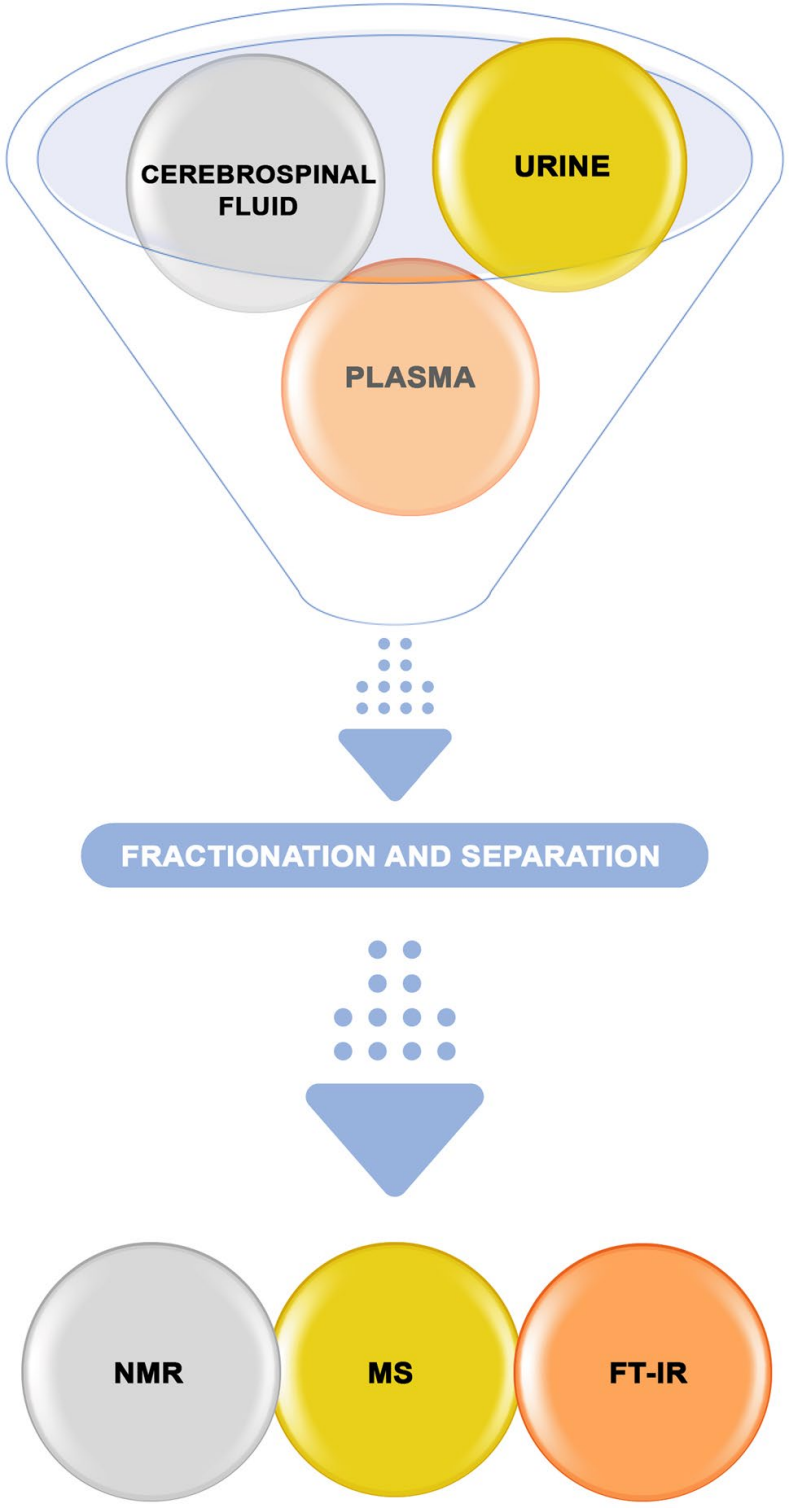
Techniques for metabolite analysis

A very similar analytical technique is capillary electrophoresis (CE) coupled with MS. CE is a relatively new separation technique that is becoming more and more common in bioanalytical science. The most serious disadvantage of CE is the detection sensitivity, which in terms of concentration sensitivity is relatively low, while the detection sensitivity in terms of mass (mass sensitivity) is very high. Slightly worse compared to high-performance liquid chromatography, mainly due to the unstable electroosmotic flow, is also the repeatability of the migration parameters (Hess et al. 2014; Koeth et al. 2013). Much greater sensitivity of the analysis is obtained using laser-induced fluorescence detection, although this method is limited only to those compounds that show fluorescence or that can be modified (derivatized) in this direction. Fourier transform infrared spectroscopy (FT-IR) measures the absorbed infrared light that passes through the sample (Fig. 2). On the basis of the spectra obtained, individual functional groups and structures are identified. In studies of metabolic profiles, FT-IR is used for complex mixtures. Identification of individual compounds is usually impossible. This method is however taken as a preliminary step to assess the differences between individual extracts. This allows us to determine which subsequent methods will be better suited for the identification of metabolites. The biological sample analyzed by infrared spectroscopy is not destroyed and can be used in subsequent studies (Kuehnbaum and Britz-McKibbin 2013; Mapstone et al. 2014).

NMR spectroscopy is a technique that uses electromagnetic radiation and the action of a constant magnetic field to determine the structure of organic compounds. NMR spectroscopy can be used to determine a large number of chemically diverse small-molecule cellular metabolites and for their structural identification. NMR is a less-sensitive technique than MS and therefore cannot be used to determine trace amounts of compounds. However, the analyzed sample is not destroyed, which enables its re-examination with the use of other techniques. The most frequently used ^1^H-NMR analysis is based on the absorption of radio frequency electromagnetic waves by proton (^1^H) nuclei under the influence of a constant magnetic field. The information obtained is displayed in the form of a spectrum, which is assigned a specific structure of the analyte in the metabolic analysis, or the spectrum is treated as a specific fingerprint of the sample (Table 3) (Martin et al. 2015; Mayers et al. 2014).

**Table 3:** Selected ^1^H chemical shifts of marker metabolites found in NMR

Metabolite	δ ^1^H (ppm)	Group	Multiplicity
Lipids	0.90	CH_3_	m
	2.00	CH_2_-C = C	m
	5.28–5.44	–CH = CH–	m
Choline-containing compound	3.21	N(CH_3_)_3_	s or m
Taurine	3.25	NCH_2_	t
	3.42	SCH_2_	t
Glycine	3.55	CH_2_	s
Glucose	4.64	1-CH	d
	5.23	1-CH	d
Polyethylene glycol	3.70	CH_2_	s
Phosphoethanolamine	3.99	OCH_2_	m

*s* – singlet, *d*—doublet, *t* – triplet, *q* – quartet, and *m*—multiplet

For NMR, minimal sample preparation is required for urine and other low-molecular-weight metabolite-containing fluids, whereas blood, plasma, and serum require extraction (using acid, acetonitrile, or two-phase methanol/chloroform protocols) or NMR-weighted techniques to separate polar and lipophilic metabolites (Table 4) (Albert and Tang 2018; Ardekani et al. 2002; Mayers et al. 2014).

**Table 4:** Biofluid and sample preparation requirements

Biofluid	Required sampling handling
Urine	Add deuterated phosphate buffer to 0.2–0.4 mL urine
Blood/plasma/serum	For 0.5 mL of heparinized blood product
	- Add deuterium oxide (to lock)
	- Add acetonitrile (for protein precipitation)
CSF	Add deuterium oxide to 0.5 mL of CSF
EPS	Add deuterium oxide to 0.03–0.10 mL of EPS
BALF	Add deuterium oxide to 0.5 mL of BALF

*CSF*—cerebrospinal fluid, *EPS*—expressed prostatic secretions, *BALF*—bronchoalveolar lavage fluid

The third key step in metabolomics research is bioinformatics data processing. The challenge for modern bioanalytics is the enormous amount of data obtained as a result of routine analytical measurements (Newgard et al. 2009). For an effective metabolomic analysis, it is usually necessary to apply bioinformatics methods in three areas: for preprocessing the obtained “input” analytical data to enable direct comparison, for searching, extracting and visualizing relationships hidden in the metabolomic data matrix, and for efficient data storage in special constructed databases (Rhee et al. 2011). Various advanced bioinformatic tools such as correlation optimized warping, dynamic time warping, Savitzky–Golay algorithms, etc. (Sansone et al. 2007).

## Analytical Strategies in Metabolomics Studies

**Fig. 3:**
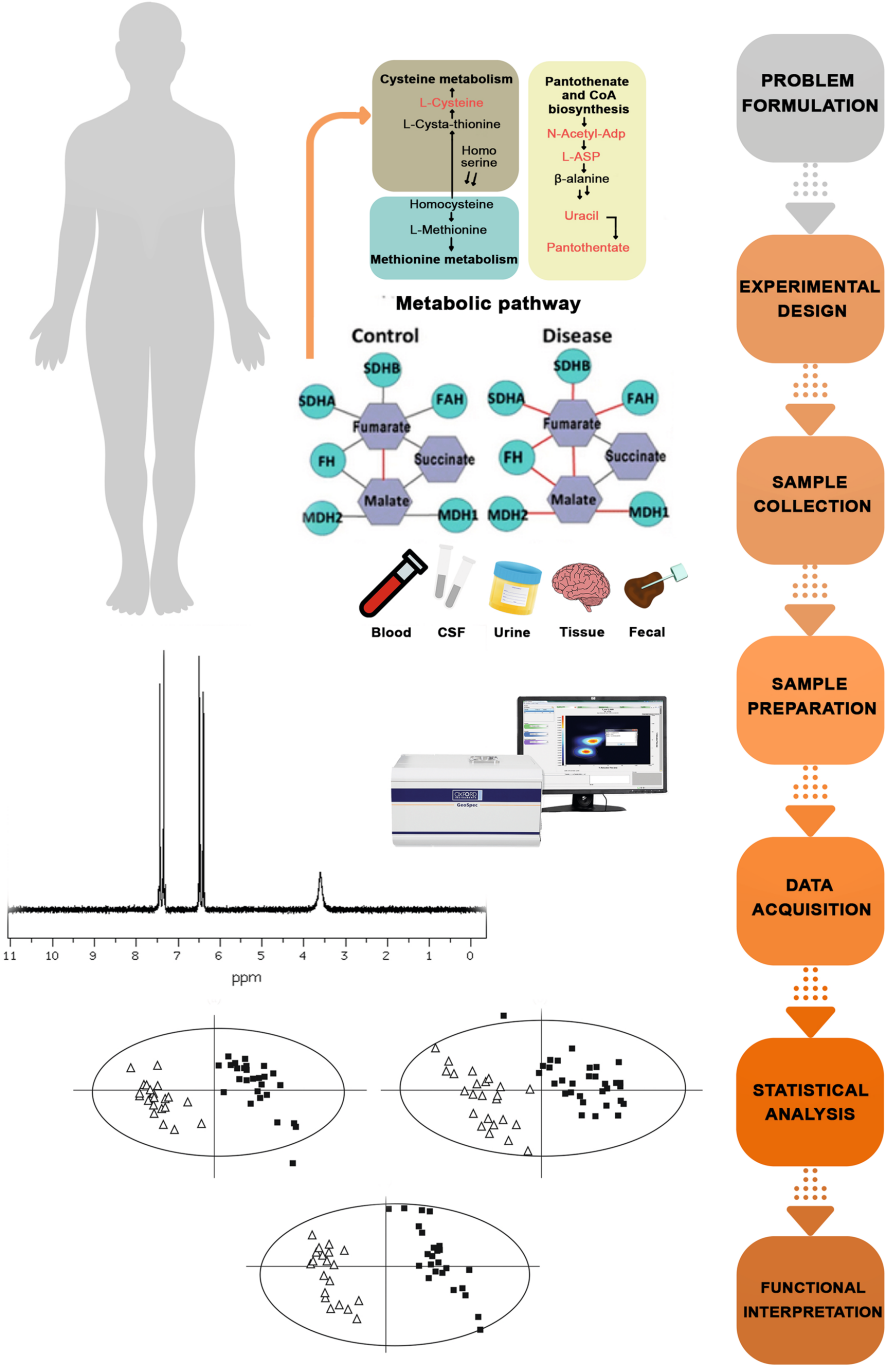
Analytical workflow of metabolomics studies

In studies on metabolites, apart from metabonomics and metabolomics, three other research strategies are clearly distinguished (Albert and Tang 2018). Metabolite target analysis is used to study the effects of changing specific factors, such as a gene mutation. A specific metabolite, being a substrate or a product of a reaction controlled by an enzyme altered as a result of the mutation, can be quantified by drawing conclusions about the effect of this mutation on metabolism. When testing a single metabolite, other coexisting metabolites that interfere with quantification can be removed during sample preparation (extraction). Appropriate sample preparation allows for increased sensitivity of analyses by eliminating the so-called noise from ballast substances. This strategy is mainly used for screening purposes and for the determination of metabolites at very low levels (phytohormones) (Albert and Tang 2018; Mayers et al. 2014) (Fig. 3).

Metabolic fingerprinting has wide application in functional genomics and clinical diagnostics. It is not always necessary to quantify the concentrations of all metabolites present in a sample. Often, a quick analysis is sufficient to allow a qualitative comparison between different samples. As a result of the comparative analysis, a classification according to the sample’s origin or biological content is obtained. A certain limitation in determining the “fingerprint” of metabolites is the difficulty to obtain repeatability of the biological sample matrix and the analytical method. Low repeatability may be a problem with the correct classification of samples and interpretation of the obtained results (Albert and Tang 2018).

The bioinformatics phase also involves the processing of the obtained analytical and clinical data using chemometric procedures to extract biologically interpreted information from the data matrix. Statistical methods such as principal component analysis make it possible to reduce large data matrices to 2–3 abstract parameters condensing relevant information. In this way, changes in the metabolic profiles, as described by many spectral variables, can be quantified. Then, by applying other computational procedures to the reduced data matrix, such as discriminant analysis or cluster analysis, the individual metabolomics study subjects are grouped and identified. Other computational methods used include partial least squares or SIMCA analysis (Sellers et al. 2015). Popular in bioanalytical science are currently the so-called artificial neural networks. In metabolomics, acute radiation syndrome is used to analyze metabolic profiles in terms of their dependence on environmental conditions (Sumner et al. 2007). An aspect of the bioinformatics analysis of metabolomics data is the appropriate visualization of the obtained results. Only after the use of effective bioinformatics tools for data visualization, it is possible to maximally simplify the comprehensive comparative analysis of multidimensional databases (van der Greef et al. 2013; Wang et al. 2011). Building reference databases of chemical compounds is very important for the correct identification of cellular metabolites. Many research teams are currently working on creating an open base of metabolites of plant or animal origin (Bai et al. 2018; Gerszten and Wang 2008; Wishart et al. 2013).

## Metabolomics as a Biomarker in Oncology: Cancer Detection

Biomarkers are widely used in clinical medicine for diagnostic, prognostic, or predictive purposes. Examples of such in oncology are the estrogen receptor. In general, quantitative metabolomic biomarkers for cancer detection and treatment evaluation are tested preclinically using animal and human cell cultures followed by validation in bioliquids or neoplastic tissue. Use of metabolomics to assess treatment effect, both as a predictive measure efficacy and as a pharmacodynamic marker, has been demonstrated in vitro studies for both traditional cancer treatments, i.e., chemotherapy and hormonal agents (Albert and Tang 2018).

The tumor metabolome begins to be characterized. Using standard metabolic methods, tumors generally exhibit elevated levels of phospholipids—characterized by an increase in total choline-containing compounds (tCho) and phosphocholine—increased glycolytic capacity, including increased use of glucose carbons to drive synthetic processes, high glutaminolytic function, and overexpression of the glycolytic isoenzyme. Among the different types of cancer, the profiles differ for many metabolites, including alanine, citrate, glycine, lactate, nucleotides, and lipids, making it difficult to generalize the results within the tumor group (Griffin and Shockcor 2004).

The National Cancer Institute, researchers and clinicians have made continual efforts expand the use of metabolomics, with a focus on magnetic resonance spectroscopy imaging for evaluation therapeutic response (Evelhoch et al. 2005). It has been found to indirectly metabolize choline phospholipids a potential biomarker for monitoring treatment efficacy in various human cancers (Glunde and Serkova 2006). Overall, the decrease in tCho signal in ^1^H-NMR corresponds to a response to chemotherapy or radiation therapy and may be an early marker of an effect as it can be detected earlier changes in conventional imaging in breast and prostate cancer, brain tumors, and other Hodgkin's lymphoma (Evelhoch et al. 2005; Griffin and Shockcor 2004).

Technical problems are also encountered in performing metabolic analyses that may hinder the characterization of the tumor metabolome, including sample variability and sensitivity, especially with extraction-dependent techniques based on multiple sclerosis.

## Cerebrospinal Fluid

Cerebrospinal fluid (CSF) plays an important role in maintaining central nervous system (CNS) homeostasis. Maintaining the appropriate volume and composition of CSF is necessary to ensure optimal conditions for the functioning of neurons (Sakka et al. 2011; Theologou et al. 2022). The cerebrospinal fluid is a water-clear, transparent fluid filling the brain's ventricles, the subarachnoid space, and the middle canal of the spinal cord. It is estimated that the daily production of fluid is approximately 500 ml, while the volume of circulating fluid in an adult human is approximately 150 ml. Therefore, the complete exchange of the CSF takes place 3–4 times/day (Theologou et al. 2022; Whedon and Glassey 2009).

It is now known that the main site of CSF production is the choroid plexuses of the lateral ventricles of the brain, and a small amount is also produced by the tissue lining the ventricles of the brain and the subarachnoid space (Brinker et al. 2014; Hajdu 2003). The resulting CSF from the lateral ventricles moves through the Monroe orifice to chamber III, then through the Sylwiusz water supply to chamber IV, to pass through the Magendi hole and through two Luschka side holes to the subarachnoid space of the brain and the spinal cord canal. The cerebrospinal fluid is absorbed by the arachnoid granules into the venous sinuses of the dura mater and then into the blood (Whedon and Glassey 2009). However, the lymphatic system is now considered to be the primary site of CSF reabsorption (Theologou et al. 2022). The formation of CSF is not a simple ul-trap plasma drainage; the mechanism of its production is more complex. Therefore, its composition is different from that of plasma (Damkier et al 2010). The first step in CSF production is the filtration of the blood plasma markers in the parenchyma of the choroid plexus, conditioned by the osmotic pressure gradient. Then, there is an active transport of ions with the participation of transport proteins in the membrane of epithelial cells of the choroid plexus (Damkier et al 2010). The composition of CSF is closely regulated, and any variation can be used for diagnostic purposes (Telano and Baker 2018).

## Use of CSF Metabolomics in Childhood Oncology

Leukemia is a group of neoplasms of the haematopoietic system and is the most common childhood cancer. The disease may be acute or chronic. Children are dominated by acute leukemias, which usually develop rapidly, much faster than in adults, and require urgent treatment. In children, the most common are acute lymphoblastic leukemias (ALL), the much rarer acute myeloid leukemias (AML), and the very rare chronic leukemias. ALL refers to hematologic malignancies of lymphoid precursor cells, which should be categorized according to the WHO classification (Swerdlow et al. 2017), and there are mainly as tumors of B-cell precursor, and T-cell lineage associated with much more favorable and worse outcome, respectively (Malard and Mohty 2020).

The symptoms of ALL in children can be non-specific, difficult to distinguish from ordinary diseases of childhood, and may last anywhere from 2 to 6 weeks until the child is diagnosed. Moreover, ALL has a significant tendency to metastasize to the central nervous system, therefore, ALL affecting children, CNS involvement at the time of diagnosis is a major clinical concern. In many cases, nondetectable CNS leukemia requires prophylactic CNS-directed conventional intrathecal chemotherapy for relapse-free survival of young patients. However, CNS-directed therapy is associated with long-term sequelae, including neurocognitive deficits and secondary neoplasms. The correct and timely diagnosis of the initial stage of the disease in the CNS remains a challenge. The sooner a correct diagnosis is made, the better the chances of recovery (Griffin and Shockcor 2004).

The cerebrospinal fluid fills the spaces in the CNS, and its composition indirectly reflects the biochemical processes taking place in the brain. Abnormal changes in certain metabolites are often closely related to the severity of the disease. The need to understand the causes of metastasis and predict their possible occurrence seems to be a priority task. The attempt to solve this problem most probably is going to be based primarily on molecular biology analysis techniques (Hess et al. 2014; Ikonomidou 2021; Kuehnbaum and Britz-McKibbin 2013). Examination of the CSF remains, despite the development of neuroimaging techniques, an essential component of neurological diagnostics. The lumbar puncture procedure allows obtaining biological material from the patient to perform specific tests and repeatedly make a final diagnosis. It also enables conducting and monitoring the effectiveness of the applied treatment, especially in infectious or inflammatory diseases. The introduction of new methods of neuroimaging resulted in a change in the strategy of diagnostic procedures and a reduction in the frequency of performing the lumbar puncture. Therefore, collecting the CSF is complicated and patients require appropriate selection (van der Velden et al. 2016).

The development of methods in the treatment of cancer has been minimized relapses outside the CNS, especially in testicular and extramedullary locations. Despite this, CNS relapses still occur in 3–8% of children with ALL or AML (Gibson et al. 2005). Endothelial blood–brain barrier (BBB), the blood–meningeal barrier, and the blood–CSF barrier are responsible for the most important for the migration of leukemia cells to the CNS (Baggott et al. 2010). Fourth the newly identified pathway is the CNS lymphatic system, which runs within the meninges and along the sinuses of the dura mater (Hockenberry et al. 2017; Zeller et al. 2014a, b). Studies in mice suggest that leukemia cells may use a shortcut along the surface of the bridging veins enter the subarachnoid space (Baggott et al. 2010). The spread of an outbreak within the CNS occurs mainly in of the meninges, may persist as focal lesions in the subarachnoid space between the arachnoid pia, and remains undetected by the lumbar puncture (Baggott et al. 2010). The standard that is used in the CNS disease assessment technology is the detection of leukemia cells in the CSF following a lumbar puncture with routine cytology (Baggott et al. 2010; Gibson et al. 2005). There are many factors that make the detection of CNS seizures difficult, including the low number of cells present in most CSF samples, presence of contaminating peripheral blood cells, and difficulties in individual interpretation of CSF cell morphology in a cytospin preparation (Berger et al. 2010; Hinds et al. 1999) (Table 5).

**Table 5:** Browsing metabolites—CSF metabolites associated

Metabolite name	Biospecimen location	Average molecular weight	Monoistopic molecular weight
1-Methylhistidine	Blood		
	CSF	169.1811	169.085126611
	Feces		
	Urine		
2-Ketobutyric acid	Blood		
	CSF	102.0886	102.031694058
	Saliva		
	Urine		
Deoxyuridine	Blood		
	CSF	228.202	228.074621504
	Feces		
	Urine		
4-Pyridoxic acid	Blood		
	CSF	183.1614	183.053157781
	Feces		
	Saliva		
	Urine		
α-Ketoisovaleric acid	Blood		
	CSF	116.1152	116.047344122
	Feces		
	Saliva		
	Urine		
p-Hydroxyphenylacetic acid	Blood		
	CSF	152.1473	152.047344122
	Feces		
	Saliva		
	Urine		
3-Methoxytyramine	Blood		
	CSF	167.205	167.094628665
	Urine		
L-Asparagine	Blood		
	Breast Milk		
	CSF	132.1179	132.053492132
	Feces		
	Saliva		
	Urine		
γ-Glutamylglutamine	Blood		
	CSF	275.261	275.111735279
	Feces		
3-Methoxytyrosine	Blood		
	CSF	211.2145	211.084457909
	Feces		
Dimethylglycine	Blood	103.1198	103.063328537
Allantoin	Blood		
	CSF	158.1154	158.043990078
	Feces		
	Saliva		
	Urine		
Myoinositol	Blood		
	CSF	180.1559	180.063388116
	Feces		
	Saliva		
	Urine		
Ribitol	Blood		
	CSF	152.1458	152.068473494
	Feces		
	Urine		

Information used in searching candidate metabolite in Human Metabolome Database (HMDB, http://www.hmdb.ca). *CSF* cerebrospinal fluid

The Brown et al.’s (2021) research group described in their work new metabolic profiles identified by GC–MS and LC–MS/MS from CSF samples taken before and during pediatric ALL therapy. They found a high concentration asparagine and γ-glutamylglutamine have been associated with intensification therapy in children with ALL. Interestingly, urinary asparagine levels have been associated with fatigue as cancer-related fatigue symptom, with higher levels reported among tired patients with breast cancer after radiation therapy and lower levels identified in people with chronic fatigue (Bartram et al. 2018; Buonamici et al. 2009; Kivisäkk et al. 2003; Oruganti et al. 2017; Svenningsson et al. 1995). Asparaginase hydrolyzes asparagine to form ammonia and aspartic acid. Leukemia cells are especially prone to asparagine depletion, because they lack sufficient asparagine synthetase activity to replenish intracellular asparagine (van der Velden et al. 2016). Aspartate synthetase catalyzes the conversion of aspartate and glutamine to form glutamate and asparagine. This amino acid is considered very important, the deficiency of asparagine synthetase expression causing dependence on extracellular asparagine pools. Since the BBB transport system regulates the concentration of amino acids in the CNS, the concentrations of asparagine in the CSF and the remaining key amino acids are approximately one-+tenth levels detected in plasma (Debik et al 2019). γ-Glutamyglutamine was first detected as widely dispersed acid dipeptide in the human brain (Buonamici et al. 2009) and varying levels of γ-glutamylglutamine in the CSF have been implicated in non-medicated schizophrenia patients (Buonamici et al. 2009) and infants with urea cycle disorders (Bartram J et al. 2018). γ-Glutamyl amino acids are intermediates in γ-glutamylcycle. Some evidence suggests that the γ-glutamylcycle of amino acid homeostasis across the BBB may be involved in regulation (van der Velden et al. 2016). The initial stage of the cycle is catalyzed by γ-glutamyl transferase, a plasma membrane-bound enzyme involved in the transport of γ-glutamyl dipeptides and glutathione across the lumen membrane of endothelial cells lining the BBB. This compound catalyzes the transfer of the glutamyl group of glutathione into an amino acid acceptor, of which *L*-glutamine is a good substrate in humans (Bartram et al. 2018). The γ-glutamyl amino acid is imported through the luminal membrane by transport systems independent of free amino acids (van der Velden et al. 2016). Intracellular γ-glutamyl cyclotransferase catalyzes the conversion of γ-glutamyl amino acids to free amino acid and 5-oxoproline, which is then hydrolyzed to form glutamate.

Studies have shown that B-cell ALL patients exhibit abnormal metabolism, including glycolysis, gluconeogenesis, amino acid metabolism, fatty acid metabolism, and choline and phospholipid metabolism. Glutamine is an essential amino acid for cancer cells. It is taken up by cancer cells as an important source of nitrogen and energy. Glutamate is also used to synthesize purines and pyrimidines to support DNA and RNA biosynthesis. To satisfy the proliferation of cancer cells, blood glutamine is depleted. Many cancers have been found to have decreased glutamine levels (Debik et al. 2019; Geck and Toker 2016; Zhou et al. 2107). In a study by the Yang team, glutamine levels increased (Yang et al. 2021). This is likely related to a lack of glutamine in the blood leading to an increase in glutamine synthase expression, and the body releases more glutamine into the blood to maintain blood glutamine levels. The study found that adult ALL patients have higher levels of unsaturated lipids, VLDL and LDL, in their plasma metabolites. This phenomenon is likely due to abnormal lipid metabolism in adult ALL patients (Wojcicki et al. 2020).

The biosynthesis of cell membranes in neoplastic tissues is very active, and a large amount of choline is required as a raw material for the biosynthesis of cell membranes. Blood levels of choline and its derivatives were elevated in many cancer patients compared with healthy controls (Christian et al. 2019; Prenen et al. 2006; Sykes et al. 2016).

Very interesting results of research using metabolomics were presented by the team of Wen-Lian Chen et al. (2014) who found that the serum patterns of metabolite changes had some similarities in patients with AML and type 2 diabetes (T2DM) compared to the control group. Except phenylalanine and tyrosine (increased in AML and decreased in T2DM), glucose, citrate, alanine, isoleucine, leucine, valine, glutamine, lysine, histidine, HDL, and choline. It is possible that the two diseases, while having a completely different pathophysiology, affect the same metabolic pathways related to glucose, amino acid and lipid metabolism. It is hypothesized that AML may interfere with insulin-mediated glucose, protein, and energy metabolism regulation. The potential prognostic power of NMR was also tested—based on metabonomics in the stratification of cytogenetic risks in AML patients. A significant phenotype related to the metabonom was found, differences between patients with AML with favorable and intermediate cytogenetic risk. However, the levels of most significantly altered metabolites were higher in patients with intermediate cytogenetic risk than in patients with favorable cytogenetic risk. Exceptions were seen for: LDL, VLDL, unsaturated fatty acids, and triglycerides, which were higher in the studies (Chen et al. 2014).

Chemical analytical methods are used as measurement methods in metabolomics, the most common of which are NMR ^1^H and ^13^C NMR spectroscopy (Deglon et al. 2012; Haug et al. 2013; Zhang et al. 2014), and less frequently ^31^P NMR (Heinrich et al. 2006; Hess et al. 2014). Another commonly used analytical technique is MS combined with LC (Koeth et al. 2013) and GC–MS (Kuehnbaum and Britz-McKibbin 2013). Due to the fact that the measurement methodology in metabolomics studies consists in comparing the changes between the group of test objects and the reference group, samples for the study of disease entities are always obtained from the group of patients, and control samples from the corresponding group of healthy people. The sources of sample origin may be very different; it is possible to use broadly understood biofluids such as serum (Mapstone et al. 2014; Zhang et al. 2014), blood plasma (Martin et al. 2015), cerebrospinal fluid (Zhang et al. 2014), urine (Mayers et al. 2014; Zhang et al. 2014), and saliva (Newgard et al. 2009).

## Conclusion

Metabolomics is a discipline that includes a comprehensive assessment of metabolites, pattern recognition, and statistical analysis. Biomarkers are widely used in clinical medicine for the prognostic or predictive interpretation of the disease state. Metabolomics should be used to identify multivariate biomarkers, including fingerprints, profiles which characterize neoplastic conditions. Using this technology, we may eventually be able to diagnose cancer earlier, while it is still recoverable, determine the aggressiveness of the cancer to help target prognosis and therapy, and predict drug efficacy. These signatures can be practical and accurate, although they also require sophisticated analytical techniques.

For other strategies currently under investigation to individualize therapy, such as assessing mutation or amplification of receptor tyrosine kinase genes, metabolomics studies should be included in preclinical and clinical studies and evaluated for the predictive value.

In fluor-deoxyglucose positron emission tomography [^18^F] FDG PET hematological malignant neoplasms unimaginable, the metabolomic analysis of circulating tumor cells after [^13^C] glucose administration can be used to evaluate treatment effects, thus providing information about the biological response non-invasively. This can also be applied to circulating tumor cells from solid tumors. While metabolomics methods have improved, and more and more evidence supports their use in clinical decision-making, the discipline is still in its infancy, and metabolomics lags behind other omics sciences somewhat due to technical constraints, database challenges, and costs. Future development and application will depend on several factors such as: establishment of spectral databases of metabolites and related biochemical identities as well as cross-validation of metabolites obtained by NMR or MS and correlation with other quantitative tests. Finally, it will be important to integrate the results of metabolomics assessment with other omics technologies so as to address the entire spectrum of malignancies the phenotype can be characterized.

Treatment of pediatric ALL remains an unresolved issue as it routinely involves CNS-directed therapy that is non-specific and may be toxic. It is extremely important to properly understand the molecular mechanisms of CNS infiltration in ALL for early recognition of the cellular features that promote cell entry into the CNS. Metabolomics appears to be an excellent solution for future research on childhood leukemia, for diagnostic and prognostic applications, all the more so as much more is known about the metabolic signatures of childhood AML than about ALL.

Appropriate understanding of the metabolic processes taking place in the human body is very important, primarily to increase the effectiveness of treatment, but also to recognize the disease in its embryo, which will certainly significantly improve this effectiveness.
